# Empowering People to Make Healthier Choices: A Critical Discourse Analysis of the Tackling Obesity Policy

**DOI:** 10.1177/10497323211027536

**Published:** 2021-06-30

**Authors:** Gavin Brookes

**Affiliations:** 1 Lancaster University, Lancaster, United Kingdom

**Keywords:** obesity, COVID-19, coronavirus, critical discourse analysis, neoliberalism, policy paper, qualitative, United Kingdom

## Abstract

In response to the heightened risk that coronavirus disease 2019 (COVID-19) poses to the health and lives of people with obesity, in 2020 the U.K. government launched a new package of policies intended to stimulate weight loss among the country’s population. In this article, I present a critical discourse analysis of the policy paper which announced these new measures. I identify the discourses that are used to represent things, people, and processes in this policy text. These discourses are interpreted in terms of broadly neoliberal ideologies of public health management. Taken together, the discourses identified contribute to a broadly neoliberal ideology of public health management. It is argued that the policy paper represents an instance of “lifestyle drift,” as it initially appears to engage with social and economic determinants of health but ultimately neglects these in favor of focusing on individual lifestyle factors, particularly in the shape of individuals’ “choices.”

## Introduction

Coronavirus disease 2019 (COVID-19) is caused by severe acute respiratory syndrome coronavirus 2 (SARS-CoV-2). On March 11, 2020, the World Health Organization (WHO) declared COVID-19 to be a global pandemic (WHO, 2020). COVID-19 can be life threatening to some demographic and clinical groups, with the group most at risk of serious illness and death from it being people aged 70 years or above. Another group that is at pronounced risk of serious complications, including death, from COVID-19 is people with obesity. The term “obesity” is a diagnostic label used to describe the condition in which a person is severely overweight and has a body mass index (BMI) score of 30 or above. Almost two thirds of adults in the United Kingdom have either overweight or obesity, with prevalence being relatively higher among people aged 55 to 74 years, people living in deprived areas, and people belonging to some minority ethnicity groups (Public Health England, 2020b). A recent review by Public Health England (2020a) of evidence on the disparities in the risk and outcomes of COVID-19 suggests that the impact of the virus has “replicated existing health inequalities and, in some cases, has increased them” (p. 4).

A review of the evidence on the impact of excess weight on COVID-19, also carried out by Public Health England, drew on findings from retrospective cohort studies, clinical audits of hospital patients with COVID-19, and routine primary care records with data linkage to patient outcomes. The report found that “excess weight is associated with an increased risk of the following for COVID-19: a positive test, hospitalisation, advanced levels of treatment (including mechanical ventilation or admission to intensive or critical care) and death” (Public Health England, 2020b, p. 6). It continues,

[t]he risks seem to increase progressively with increasing BMI above the healthy weight range, even after adjustment for potential confounding factors, including demographic and socio-economic factors. There is also some evidence to suggest that disparities in excess weight may explain some of the observed differences in outcomes linked to COVID-19 for older adults and some BAME [Black, Asian, and Minority Ethnic] groups. (Public Health England, 2020b)

The report also indicates a possible interaction between weight-related comorbidities, such as type 2 diabetes and cardiovascular and respiratory diseases, which are linked to more severe cases of COVID-19, in addition to certain socioeconomic and demographic factors that have been linked to both excess weight and COVID-19 risk. In response to the link between obesity and the virus, on July 27, 2020, the Government of the United Kingdom published a policy paper, titled *Tackling Obesity: Empowering Adults and Children to Live Healthier Lives* (Department of Health & Social Care, 2020), which set out a “new obesity strategy to get the nation fit and healthy, protect themselves against COVID-19 and protect the NHS.”

In this article, I subject this policy paper to a critical discourse analysis (CDA; Fairclough, 1995/2010, 2015). In particular, I critically examine text designers’ linguistic choices with respect to the ways in which social actors and processes are represented. These linguistic choices are interpreted in terms of the discourses that they encode which are, in turn, interpreted as supporting particular ideologies around obesity and health, while backgrounding others. For this purpose, I take a broadly social constructionist view of discourse and follow Burr (1995), who defines it as

a set of meanings, metaphors, representations, images, stories, statements and so on that in some way together produce a particular version of events [. . .] Surrounding any one object, event, person etc., there may be a variety of different discourses, each with a different story to tell about the world, a different way of representing it to the world. (p. 48)

This view of discourse is grounded in poststructuralist theory and in particular the writings of Foucault (1972). Ideologies are understood to be “ways of representing aspects of the world, which may be operationalized in ways of acting and interacting and in ‘ways of being’ or identities, that contribute to establishing or sustaining unequal relations of power” (Fairclough, 1995/2010, p. 8). In this article, I attempt to show and critically assess the understandings that underpin the discourses that are used to represent the obesity policy.

Following this introduction, in the “Obesity Policy in the United Kingdom (in a COVID-19 Context)” section, I describe in more detail the public health policy context in which the present study and its data are situated, before providing a more detailed account of my data and methodological approach in the “Method” section. The “Findings” section reports the findings in terms of the discourses that characterize the policy paper. These discourses are then discussed and connected to wider obesity- and health-related ideologies in the “Discussion” section. The “Conclusion” section then summarizes the study’s main findings, considers their implications for people with obesity and public health more broadly, and gestures to avenues for future research on this topic.

## Obesity Policy in the United Kingdom (in a COVID-19 Context)

To critically engage with policy intervention relating to obesity, it is necessary to examine what Mulderrig (2019b) aptly describes as “the contested terrain of obesity knowledge that shapes the ‘landscape of assumptions’ which underpin governmental and public perceptions of obesity as a societal problem” (p. 103). A measure of the degree of consensus on the risks that obesity poses to society, she argues, is that it is “now commonplace for governments, health organizations, and the media to talk about the ‘obesity epidemic’” (Mulderrig, 2019b; see also Boero, 2007). Indeed, this militaristic metaphor also seems to have gained traction in mass media reporting of obesity (Brookes & Baker, 2021). This metaphor can be viewed as an attempt to capture obesity’s purportedly rising rates of incidence throughout much of the developed world (Boero, 2007). The “epidemic” metaphor also encapsulates the health risks that have been attributed to obesity, which include increased risk of certain chronic diseases as well as reduced life expectancy overall (Public Health England, 2020b).

A consequence of the “epidemic” trope is that it invokes causal links with so-called “life-style diseases” and treats excess weight as a disease itself (Mulderrig, 2017). The dominance of the understanding of obesity as disease (Lupton, 2018) may be said to contribute to the backgrounding or suppression of seeing obesity in conjunction with being a symptom of social inequality. As Bissell et al. (2016) argue, obesity demonstrates a well-established social gradient in prevalence, with the highest rates found among those who are most socioeconomically disadvantaged (see also Ulijaszek, 2014). Rates of obesity also exhibit a relationship to food poverty, as measurable through the use of food banks. Bissell and colleagues point out that evidence increasingly points to “material lack and precarity which are increasingly features of daily life across many countries,” with “rising levels of material and financial hardship [. . .] clearly impact[ing] the food decisions of many” (Ulijaszek, 2014). It is with all this in mind that Marsh (2004) argues obesity to be a “symptom of social impoverishment.”

U.K. Government policies around obesity, however, have tended to focus more on ways to persuade individuals to modify their behaviors and lifestyle choices to reduce their personal obesity “risk,” especially through modifications to their diet and exercise habits. An example of this is the government’s *Change4Life* campaign. Established in 2009 by Public Health England, *Change4Life* is the United Kingdom’s first national social marketing campaign designed to address the causes of obesity. Its objective is to persuade individuals and families to implement small and sustainable changes to their diet and activity levels, as indicated in its slogan, “eat well, move more, live longer” (Sweeney, 2008). (Critical) studies of the discourses utilized as part of this and other campaigns have identified the linguistic mechanisms by which this individualizing, neoliberal perspective on health is realized and members of the public accordingly responsibilized for their well-being. For example, across a series of articles, Mulderrig (2017, 2018, 2019b) investigated the use of “nudge” tactics in the *Change4Life* campaign, whereas Brookes and colleagues examined the use of multimodal discourses as part of emotional appeals and persuasion tactics in U.K. public health campaigns around diabetes (Brookes & Harvey, 2015), mental health (Brookes & Harvey, 2016a), baby-feeding practices (Brookes et al., 2016), and dementia (Brookes et al., 2021).

(Critical) research of the discourses that characterize public health texts in the United Kingdom, including those mentioned above, has interpreted the use of responsibilizing rhetoric in these contexts as evidence of the wider influence of neoliberalism in British society. Neoliberalism is a theory of political economic practice which proposes that human well-being is best advanced by liberating individual entrepreneurial freedoms within a free market institutional framework (Harvey, 2005). The role of the state in this context is to create and maintain this institutional framework to support such practices. Mulderrig (2019a) describes how a cross-party consensus on neoliberal modes of governance has been maintained, in various forms, in the United Kingdom since the 1980s. The shift toward, and maintenance of, neoliberal modes of governance has involved a necessary reconfiguration of power relations, whereby the locus of responsibility for health and well-being is shifted *away from* the state and instead placed within the individual citizen-consumer. According to this model, not only ill-health but also social ills like unemployment and poverty are framed as risks that individuals are responsible for managing and, as such, for which they can be assigned blame if and when that risk materializes. As a consequence, governments “develop various forms of intervention designed to steer individuals towards ‘appropriate’ or ‘desirable’ outcomes, and in doing so diagnose social problems as a problem of self-government rather than of capitalism, racism, inequality, and so on” (Mulderrig, 2019a, p. 51; see also Brown & Baker, 2012; Harvey, 2005).

Critical explorations of the discursive means through which neoliberalism is enacted in public health and healthy policy texts have drawn fruitfully upon the concepts of responsibilization and governmentality. Responsibilization refers to the process by which individuals are transformed self-governing citizens who assume full responsibility for their lives and actions (Burchell, 1993). Grey (1997) argues that responsibilization is about rendering individuals as “trustworthy and predictable by virtue of their beliefs and behaviors” (p. 719), whereas Brown and Baker (2012) add that the key to this process is “giving people knowledge or information as their initiation into some sort of technical expertise” but point out that “it is not because this knowledge necessarily assists their working or personal lives but it is part of a Foucauldian process of rendering them docile” (p. 18). It is in this sense that responsibilization aligns with governmentality—a concept deriving from the writing of Foucault (1991) which refers to “a form of political power comprising a range of technologies, mentalities and rationalities of governing others and oneself” (Brown & Baker, 2012, p. 18). It involves “acting on the manner in which individuals regulate their own behaviour” (Hindess, 1996, p. 106). Through the process of responsibilization, the neoliberal state encourages or compels its individual citizen-consumers to manage their risks in the ways in which they exercise their increased freedoms, thereby effectively self-governing. The role of the government is therefore minimized in this context, and reduced to encouraging and imploring practices of self-management and establishing the rules and boundaries within which such activity takes place.

It was against this backdrop, then, of neoliberal, responsibilizing public health policy around obesity that COVID-19 struck, and the U.K. government formulated a fresh policy response to obesity. This came in the shape of the *Tackling Obesity: Empowering Adults and Children to Live Healthier Lives* policy paper. The paper promises a package of measures designed to reduce the prevalence of obesity in the country, including the following:

- Expanding NHS England weight services;
- Legislating for mandatory calorie labeling on food and drink items in cafes, restaurants, bars, and takeaways in businesses with more than 250 employees;
- Legislating to restrict supermarket promotions on foods that are high in fat, salt, and sugar;
- Introducing new laws banning online and televised advertising of such food products before 9:00 p.m. (when children are more likely to see them);
- Launching two new consultations on front-of-pack nutrition labeling and calorie labeling on alcohol;
- Launching a new Public Health England campaign, *Better Health—Let’s Do This!*, which includes a website and smartphone app designed to help people to lose weight.

Public health authorities in the United Kingdom are no stranger to campaigns intended to instigate weight loss in the population. So, why this campaign, now? One argument is that the pandemic has brought to the fore the health complications associated with obesity and as such may be perceived by policymakers and government advisors to constitute a “teachable moment” for health behavior change (Lawson & Flocke, 2009)—an opportune time at which to address what is, in obesity, a long-term and ongoing public health issue. The policy paper itself describes COVID-19 as a “wake-up call” with respect to the health impacts of obesity, while the British Nutrition Foundation (2020) comments that the “health risks associated with obesity have been brought into sharp focus by the coronavirus pandemic.” There is also evidence that the measures have been put in place as a response to a perceived threat that people with obesity pose to the country’s National Health Service (NHS; Campbell et al., 2020).

While the new policy paper and the package of measures it promises have been widely welcomed, they have also received some criticism, for example, from organizations like the British Nutrition Foundation who, though welcoming the strategy overall, also stated that

[t]he support for people who want to lose weight from the NHS England and PHE initiatives is welcome. However, given the scale of the problem, it is likely that further action across many different areas will be needed. This includes tackling the socioeconomic inequalities that we know are associated with risk of obesity, especially in light of the serious economic effects of the COVID-19 outbreak. (Greenhill, 2020)

The strategy was also criticized in an editorial in *Nature Reviews Endocrinology*, which argued that the campaign focused too much on reducing calorie intake and missed the opportunity to educate people on what a balanced diet involves, as well as implementing measures such as making nutritious foods more affordable, expanding the soft drinks levy to include foods that are known to be detrimental to health, and tackling the so-called “obesogenic environment” by making “active commuting” easier and safer, limiting the number of fast-food outlets near schools, and improving access to green spaces (Greenhill, 2020). Criticism has also come from some sections of the (liberal) media, who argue that the measures fail to address underlying inequalities that mean that people from lower income backgrounds are more likely to eat cheaper and filling but nutritionally poor food—for example, in an article in *The Guardian* titled “New UK Obesity Plan Fails to Address Underlying Problems” (Boseley, 2020)—which criticized the strategy for not doing enough to promote “healthy” food (in addition to placing constraints on the promotion of “unhealthy” food), as well as making these types of food more available, for example, through government subsidies. As well as its effectiveness from a public health perspective, critics have also drawn attention to the potential for the suddenness of the campaign to make individuals with obesity feel personally targeted by it (Littlewood-Hillsdon, 2020).

Many of the criticisms directed at the campaign echo those that have been leveled at previous health campaigns in the United Kingdom, as noted earlier. Compared with the *Change4Life* campaign, this new package of policies is more wide-ranging, being more concerned than its predecessor with legislative change, increased taxation on particular products, and the tightening of regulations around advertising. In other words, on the surface level at least, this campaign seems to go further than the pleas to individual accountability which underpinned the *Change4Life* campaign and others like it. By critically examining the discourses and ideologies that characterize the *Tackling Obesity: Empowering Adults and Children to Live Healthier Lives* policy paper, the present study will be well positioned to compare this new campaign against previous ones on the basis of findings from studies of such campaigns which have employed similar, (critical) discourse-based analytical methods. It must be noted that it is not the aim of this study to examine the policies themselves but, rather, to identify and critique the linguistic choices and discourses through which the policies and the various social actors involved are represented, and to consider the health-related (and other) ideologies that these discourses may encode. To my knowledge, this is the first study to examine the discourses that constitute this policy paper. Yet, the discourses that constitute policy documents such as this are certainly worthy of critical scrutiny, as discourses play a key role in constructing problems and *legitimating* particular responses and courses of action in this context. Put simply, legitimating discourses are those discourses which provide the answer to the question “Why?”—“Why should we do something, and in a particular way?” (van Leeuwen, 2007). Mulderrig et al. (2019) characterize policy as resting on “political imaginaries” and describe how they “construct a particular version of the problem, legitimated on the basis of available expert evidence, and are shaped by the dominant mode of governing,” whereby the “language of policy plays a significant role in conceptualising the policy problem in specific ways and in legitimating the solution(s) it proposes” (p. 6).

## Method

As noted, the data examined in this study is the *Tackling Obesity: Empowering Adults and Children to Live Healthier Lives* policy paper. The paper was published online on July 27, 2020, on the website of the Department of Health and Social Care (2020). The policy paper was downloaded and analyzed using a CDA approach (Fairclough, 2015). CDA is an approach to discourse analysis (Cheek, 2004), which synthesizes close analysis of linguistic choices with theoretically informed accounts of context to elucidate how discourse produces and reproduces social practices and legitimizes certain ways of acting and being over others. From this perspective, it is through language and discourse that social problems like obesity are constituted and contested, and thus social change is accomplished. CDA is an interdisciplinary research movement (van Dijk, 1995) that comprises a range of analytical models. Approaches to CDA can be distinguished from more traditional discourse analysis (see Starks & Brown Trinidad, 2007) in the sense that they are united by a focus on the discursive dimensions of power and social justice, and as such share an explicitly problem-oriented, emancipatory agenda.

The analytical approach taken in this study orients most closely to the dialectical–relational approach to CDA developed by Fairclough (2015). This approach is underpinned by a dialectical–relational view of discourse, from which discourse is understood both be constitutive of and constituted by social practices. The aim of CDA from this perspective is to not only describe and critique discourses but to also explain the social and ideological conditions which both give rise to those discourses and are enabled by them.

On a practical analytical level, a discourse can be identified through the “patterned use of language which emerges from engagement in social practices” (Mulderrig et al., 2019, p. 11). Likewise, Mills (1997) states that discourses can be identified through “the systematicity of the ideas, opinions, concepts, ways of thinking and behaving which are formed within a particular context” (p. 17). I identified discourses through qualitative analysis of recurring linguistic choices in representations of (a) obesity, (b) citizens, and (c) the government, including the actions, attributes, and values that were ascribed to them. In particular, I focused on choices pertaining to lexis (e.g., nouns, main verbs, adjectives, adverbs), grammar (e.g., active and passive voice), as well as the use of pronouns and rhetorical devices such as metaphorical and vague language. Metaphor is broadly the phenomenon whereby one thing is spoken about (and potentially thought about) in terms of another. Vague language is a form of semantic indeterminacy which can be identified on the basis of two criteria: (a) that it is at least theoretically possible to express the utterance more precisely and (b) that the indeterminacy of the expression must arise from the linguistic expression (Pinkal, 1995).

Recurring representational (linguistic) choices are interpreted as constituting discourses, which are in turn linked to particular functions (e.g., assigning responsibility, foregrounding or backgrounding factors, legitimating particular actions) and the ideologies that underpin and are espoused by them (e.g., neoliberalism, governmentality).

## Findings

The analysis is divided into three sections which broadly represent the discourses that are used in relation to the three areas of representation noted above: (a) obesity, (b) citizens, and (c) the government. In relation to (a), I demonstrate how obesity is discursively conceptualized as a threat that needs to be countered. In relation to (b), I show how citizens are positioned as consumers who are responsible “choice-makers.” Finally, with respect to (c), I demonstrated how the government is constructed as a benevolent social actor that helps citizen-consumers to help themselves. These discourses each result from a culmination of particular lexical and grammatical choices made to represent the various things, people, and processes that are in some way implicated in the policies being introduced. The discourses are explored separately for the facility of analysis. However, they relate to overlap and relate to each other both in the policy text itself and in terms of their likely effects. This interconnectedness will be explored in the “Discussion” section. I should also note that the sections are imbalanced in size and that this imbalance reflects the fact that some areas of analytical focus were discussed more than others in the policy text, leading to more and more diverse forms of representation. This imbalance thus reflects the nature of the policy paper data.

### Conceptualizing Obesity: A Threat to Be Countered

The first set of representations, or discourses, that I want to consider construe obesity as a problem or threat. Perhaps the most familiar representation which contributes to this discourse is that of obesity as something that poses a threat to human life. This includes forging a link between obesity and other health problems.

(1) Obesity is associated with reduced life expectancy. It is a risk factor for a range of chronic diseases, including cardiovascular disease, type 2 diabetes, at least 12 kinds of cancer, liver and respiratory disease, and obesity can impact on mental health.

Notably, the health risks that are linked to obesity, as in the above example, are presented as noun phrases, rather than being rendered as processes. For example, rather than being framed as causing the process “dying,” obesity is “associated with reduced life expectancy.” Likewise, rather than obesity causing someone to “develop or experience mental illness,” readers are informed that “obesity can impact on mental health.” Such linguistic choices help to construct rather vague relationships between obesity and health problems (e.g., “associated with,” “can impact on”). In each case, the precise nature of the relationships between obesity and these health problems is obfuscated. This perhaps reflects that these relationships are, in fact, contested and comprise a range of “knowledges,” some of which posit that obesity does not actually cause these health problems but may, for example, co-occur alongside obesity but in fact result from other factors that they have in common with obesity (see Lupton, 2018, for a review).

Another effect of these relationships being underspecified is that the individuals concerned become obfuscated, meaning that obesity can be interpreted as a threat to the health of all, not just people living with it. This is explicit in the construction of obesity as “one of the great health challenges of our age”—another nominalizing construction which discursively collapses the various and complex processes and factors that underpin obesity into a “health challenge”—a “great” one at that—and one that is owned (*our*), and should be addressed, by all in society. Generalizing the threat of obesity in this way helps to contribute the sense in which it indeed constitutes a crisis or epidemic—as discussed earlier.

Obesity’s threat is not only relevant to the current generation but is extended into the future, as threatening the health of children, particularly as they enter adulthood.

(2) Our country’s rates of obesity are storing up future problems for individuals and our NHS.
(3) Today, around two thirds (63% of adults) are above a healthy weight, and of these half are living with obesity. We have one in three children leaving primary school who are already overweight or living with obesity with one in five living with obesity.

As the above example indicates, as well as constituting a threat to the nation’s health, obesity (and specifically the demands of providing obesity-related treatment) is represented as a threat to the NHS. This discourse is particularly pervasive across the policy paper, wherein obesity is framed as putting pressure on health care services.

(4) Obesity has become an immediate concern for anyone who is overweight and for our health and care services.
(5) Obesity puts pressure on our health service. It is estimated that overweight- and obesity-related conditions across the United Kingdom are costing the NHS £6.1 billion each year.

These discourses are, in many ways, familiar by now, as they reflect broader dominant discourses around obesity in British society, as illustrated, for example, by studies identifying their prevalence in mass media (Brookes & Baker, 2021). From a policy perspective, this may pose the question: Why a new policy, now? The threat that obesity is presented as causing to the country’s health and its NHS is, as such, framed as having been intensified by COVID-19. This is evaluated as “worrying” and as making the general, aforementioned “challenge” posed by obesity “all the more important.”

(6) But worryingly, there is now consistent evidence that people who are overweight or living with obesity who contract coronavirus (COVID-19) are more likely to be admitted to hospital, to an intensive care unit and, sadly to die from COVID-19 compared to those of a healthy body weight status.
(7) Obesity is one of the great health challenges of our age; COVID-19 has made this all the more important.

The point of constructing obesity as a threat, to individual and collective health and the NHS, and a threat that has been intensified by COVID-19, is that it serves to legitimate the need for actions that are taken to counter this threat. These actions are frequently framed as metaphorical battles or struggles—a choice of trope which helps to further construe the sense of obesity as a (violent) threat that needs to be repelled. For example, the policy paper itself is titled “*Tackling* Obesity,” while the paper argues elsewhere that “*tackling* obesity is one of the greatest long-term health challenges this country faces.” The example below provides another example of this metaphor, in which this relationship between obesity threat and particular courses of action designed to counter it is construed explicitly.

(8) Tackling obesity would reduce pressure on doctors and nurses in the NHS, and free up their time to treat other sick and vulnerable patients. If all people who are overweight or living with obesity in the population lost just 2.5 kg (one third of a stone), it could save the NHS £105 million over the next 5 years.

This example demonstrates another discourse that underpins many of the assumptions operating throughout the policy paper; namely, that obesity is an avoidable health problem. This is implied through the presentation of weight loss as an endeavor which requires will on the part of individuals (“if all people who are overweight or living with obesity . . . lost just 2.5 kg”), where the use of *just* minimizes the amount of effort that is implied to be necessary. This construction also implies a hierarchy of conditions where other, unspecified conditions (presumably ones which are not considered to be avoidable) are implied to be more deserving of doctors’ and nurses’ time. This echoes discourses, observed in previous research, wherein treatment for so-called “lifestyle” diseases, which are perceived as being self-inflicted, are positioned as being less deserving of medical support compared with other, “non-lifestyle” diseases (e.g., comparisons between obesity treatments and treatments for cancer—see Brookes & Baker, 2021).

Another feature demonstrated by the above example is that “tackling obesity” is legitimated on the basis that it would alleviate pressure on the NHS, which is referred to metonymically in terms of the specific types of health care workers—“doctors and nurses.” Of course, the range of health care staff that would be involved in provision of care to people with obesity (and others) is, in reality, much broader. However, this reference to doctors and nurses in particular helps to personalize the appeal of the message; that is, that reducing rates of obesity will free up time for the treatment of other (presumably more important or deserving health problems). This emotive appeal is evident elsewhere in the text, particularly where weight loss, and the measures set out in this policy paper, is framed as being for the good of the NHS.

(9) We owe it to the NHS to move toward a healthier weight.
(10) Going into this winter, you can play your part to protect the NHS and save lives.

As these examples attest, not only are actions intended to counter the threat of obesity framed as being for the good of the country and the NHS, but through this the stakes are raised, with individuals (readers) presented having an obligation to the NHS “we *owe* it to the NHS” and to protect the NHS and others’ lives. These examples, and the second one in particular, also gesture toward a set of neoliberal discourses which personalize responsibility for managing obesity risk and reversing obesity by placing it with individuals (i.e., *you* can play *your* part to protect the NHS and save lives). These discourses are explored in the next section, which considers the construction of citizen-consumers as responsible for developing and eradicating obesity.

### Assigning Responsibility: Constructing the Citizen-Consumer and Foregrounding Choice

The next set of representations that I want to consider contributes toward a neoliberal, responsibilizing discourse around obesity which construes it as something that results from individuals’ (read: citizen-consumers’) lifestyle choices, a corollary of which being that obesity can thus be eradicated by citizen-consumers making better choices about how they live their lives. This discourse is evident throughout the policy paper but is particularly visible in descriptions of obesity as “modifiable.”

(11) Excess weight is one of the few modifiable factors for COVID-19.

This discourse is also evident in cases where a parallel is constructed between obesity and behaviors that are perceived as modifiable lifestyle choices, such as smoking. In this case, the policy paper promises to ‘learn lessons’ from the interventions that were previously developed to discourge smoking among the public, presumably to implement these in the context of obesity prevention and treatment.

(12) This includes learning the lessons from smoking, where GPs (General Practitioners) played a key role in raising the topic and doing behavioral interventions, including referrals to stop-smoking services.

If obesity does, as the policy paper suggests, result from lifestyle choices, this text also makes it clear that these choices relate to diet and exercise, but particularly the former, by drawing on what Crossley (2004) describes as the “energy equation” explanation of obesity—that is, that obesity results when individuals consume more calories than they expend.

(13) As a nation, we are eating and drinking too many calories. Many adults are consuming 200 to 300 extra calories a day and children who are already overweight or living with obesity are consuming up to 500 calories extra. We need to make sure that across the nation, we do not take in more calories than we need. But we need to make it easier.

From this perspective, obesity relates to individuals making “unhealthy” choices with respect to their diet and exercise, but particularly the former. The concept of “choice” is key to this discourse, and the lemma CHOICE occurs 18 times throughout the text, consistently in contexts where measures to eradicate obesity are framed as individuals making better/healthier choices.

(14) We know that people would welcome more support to make healthier choices for themselves and their families when eating out, with clear information about calorie content to make informed decisions.
(15) It is hard to make the healthy choice if you do not know what is in the food you are eating. That is why we want to make sure that our labeling of products in store and in cafes and coffee shops helps us to make healthier choices. We know that when shopping, identifying the healthiest products is not always easy. We want to do all we can to help people wherever they shop, to make informed food and drink choices that help them improve their health.
(16) It is fundamental that we all have access to the information we need to support a healthier weight, and this starts with knowing how calorific our food is. We are used to knowing this when we are shopping in the supermarket; most of the food and drink are clearly labeled in some form with calorie information, but there is often a lack of information when we eat out or get a takeaway.

The concepts of “choice” and self-determination are at the heart of neoliberal politics (Brown & Baker, 2012; see also Rose, 1990/1999). Examples (14) to (16) reveal two further interesting features that are relevant to the discourse explored in this section. The first is the construction of information and knowledge as being fundamental to making good choices. Unhealthy choices are thus equated with a lack of knowledge, in this case about food and calorie content: “It’s hard to make the healthy choice if you don’t know what’s in the food you are eating,” “We know that when shopping, identifying the healthiest products is not always easy.” The difference between making healthy and unhealthy choices is thus construed as the difference between having and not having this knowledge and information, and the text indicates that policies will be geared toward providing citizen-consumers with that knowledge and information to enable them to make “healthier” choices: “We know that people would welcome more support to make healthier choices for themselves and their families when eating out, with clear information about calorie content to make informed decisions”; “we want to make sure that our labelling of products in store and in cafes and coffee shops helps us to make healthier choices”; “We want to do all we can to help people wherever they shop, to make informed food and drink choices that help them improve their health”; “It is fundamental that we all have access to the information we need to support a healthier weight, and this starts with knowing how calorific our food is.”

The second feature of Examples (13) to (15) that is worth noting is the rather explicit construction of the individual (including imagined readers) as consumers. In these examples, this is a rather literal representation, as the food choices being made are all in the context of purchasing food in supermarkets, restaurants, or takeaways. In these examples, the citizen-consumers are referred to as “people” or are included in the collective “we” (more on this later). However, at other points, the positioning of citizens as consumers is rendered even more explicitly, for example, in cases where references to citizens “functionalize” them by denoting them in terms of their roles as “customers” (see Examples (27) and (28) further down) and “consumers” (below).

(17) Therefore, we will consult before the end of the year on our intention to make companies provide calorie labeling on all prepackaged alcohol they sell, so when consumers shop for alcohol, they have all the information they need to make healthier choices.

This positioning is also evident in pledges, in the text, that citizen-consumers will be given a “fair deal”—a metaphor which positions situates citizens in a transactional context by drawing on lexis which frames them, for example, as being involved in a “deal.”

(18) That’s why when it comes to food and drink, we want to ensure everyone has the right information, that they are offered a fair deal and that they are not unduly influenced to purchase less healthy foods and drinks.

The construction of citizens as self-determining consumers is fundamental to neoliberal models of governance. This is because, as Brown and Baker (2012) point out,

[t]he degree of self-reflexivity involved in the construction of identities has been enhanced through the undermining of traditional forms of expertise and the development of consumer culture. Individuals are presented with ever more diverse forms of knowledge, expertise and authority from which to choose. (p. 13)

This was not the only metaphor used to represent citizen-consumers, and the other tropes drawn upon in this text also contribute to the individualizing, neoliberal discourse. One such example is in cases where citizen-consumers are framed as engaging in lone weight loss “journeys.”

(19) We will be introducing a new campaign—a call to action for everyone who is overweight to take steps to move toward a healthier weight.

So far, this section has demonstrated how obesity is represented as resulting from individual citizen-consumers’ lifestyle choices, and so as something that can be avoided or eradicated through those individuals making “healthier” choices in their lives, with particular emphasis placed on the food that they buy and eat. These representations are individualizing in the sense that they foreground the behaviors and choices of individuals to account for how obesity develops and, in turn, how it should be countered. An effect of this foregrounding is that other, social and economic factors underlying obesity are backgrounded. Inspecting the policy text further, it emerges that such factors are indeed backgrounded through the linguistic choices that inform their representation, as in the example below.

(20) Obesity prevalence is highest among the most deprived groups in society. Children in the most deprived parts of the country are more than twice as likely to be obese as their peers living in the richest areas. This is sowing the seeds of adult diseases and health inequalities in early childhood.

In this example, obesity prevalence is framed as being most prevalent in “the most deprived groups of society.” However, rather than use this opportunity to discuss the social inequalities that underpin this trend, or to introduce policy that targets this inequality explicitly, this trend is instead reported in a way in which agency (i.e., the groups or individuals responsible for this inequality) is omitted. Even the choice of “the” in “the country” can be contrasted against “this country” or “our country” (used elsewhere in the policy text—see earlier, including Example (2)) as it creates distance between the country and the government/voice of the policy paper. This is perhaps because the government would prefer not to claim ownership of the country in a context of discussion around social inequality and its effects on obesity rates. In this vein, it is also notable that these children are not depicted as being possessed, in contrast to other points in the paper (e.g., in the section titled, “Shaping the Marketing to *Our Children*”).

Another way in which agency is concealed in relation to social inequality is through the use of metaphor. As seen previously, the policy paper makes frequent use of militaristic metaphors to construct the country as being at war with obesity and individuals as being engaged in struggles or journeys which represent their weight loss attempts. Example (20) is notable for its use of a plant metaphor, with childhood health inequalities framed as “sowing the seeds” of diseases in adulthood. Within this metaphor, the people and things that are responsible for creating and maintaining health inequalities are elided, with inequality instead construed as an organic being which thus takes on a life of its own. From this view, it is the growth of the seeds that results in health problems in adulthood, rather than the causes of systemic childhood health inequalities, that are in focus. A similar phenomenon was reported earlier in this article, where rates of childhood obesity were again depicted using a depersonalized container metaphor, being framed as “storing up” problems for the future (see Example (2) earlier), where again the agent who “stores up” health problems is the rate of childhood obesity, which is anthropomorphized in this case.

Another factor that is acknowledged as playing a role in the development of obesity, but which is again mitigated, is advertising and marketing of calorie- and sugar-laden food and drink. As well as again reducing the focus on obesity causes to individuals’ eating and drinking habits, there is also a recurrence of the focus on individuals’ choices being problematized in the face of such advertising.

(21) Many people have tried to lose weight but struggle in the face of endless prompts to eat—on TV and on the high street. In supermarkets, special offers and promotions tempt us to buy foods that are not on the shopping list but are hard to resist.
(22) We know that people want more choice on offers that support a healthy lifestyle and that high fat, salt, and sugar (HFSS) products displayed prominently at shopping tills or on the end of aisles tempt us to add extra items to our baskets and “pester power” from children puts pressure on parents to purchase unhealthy items at the shopping till.

In Example (21), weight loss is framed as a “struggle” in which “many people . . . face endless prompts to eat.” While the site of the advertisements are provided (TV and the high street), the social actors (supermarkets, marketers, agencies) who put them there are obscured. In fact, it is the advertisements and promotions themselves which are anthropomorphized as “tempt[ing]” us to purchase food in both examples. Such lexical choices muster the incorporation of Protestant ethical values in debates around obesity, observed by Flint et al. (2016) in media reportage, according to which obesity comes to be viewed as a form of deviant and immoral behavior that emerges from the failure of individuals to exercise restraint by avoiding temptation.

Example (22) usefully illustrates how the agency around advertising creation is obscured, where the location of point-of-sale advertising is foregrounded (being agentlessly “displayed” at shopping tills and aisle ends) in contrast to “pester power” which is clearly attributed to “children” who are accordingly blamed for putting parents under pressure to purchase “unhealthy items at the shopping till.”

This problematization of food and purchasing *choices*, as opposed to the advertisements and promotions themselves, is also evident at other points in the text—for instance, in this example in which advertising is relegated to an ancillary role, as “shap[ing] and influenc[ing]” individuals’ “food choices.”

(23) we know our food choices are shaped and influenced through advertising in its many forms.

Thus, making “healthy” choices when shopping is all about having the “right information” in face of food advertising. This is the culmination of the representations analyzed in the foregoing section and is demonstrated by the following example.

(24) The right information is important, but the deals and offers presented to us when we are shopping play an undue role in shaping our choices. These promotions are a key part of our shopping experience, but often these are not fair and present us only with unhealthy options.

At other points, the “struggle” becomes so individualized that it is implied to involve individuals battling their “biological programm[ing]” in the face of advertising.

(25) We are biologically programmed to eat and when we are bombarded by advertisements and promotions for food, it is hard to eat healthily, especially if we are busy or tired or stressed.

This example also attests how it can be the circumstances of individuals’ lives—namely, their business or stress—that are problematic, again rather than the advertising itself. This example also demonstrates another way in which passive voice constructions are used to obscure reference to the social actors and organizations responsible for this advertising and promotion; “we are bombarded by advertisements and promotions for food,” though readers are not told who created those advertisements and who benefits from them.

This does not mean, though, that supermarkets were not mentioned in the policy text at all. However, references to these social actors can be confusing as the referents of pronouns switch within the space of sentences and paragraphs. For instance, in Example (13) analyzed earlier, the first-person plural form, *we*, has two different referents within the space of consecutive sentences, switching from the population of the country, “*We* need to make sure that across the nation *we* don’t take in more calories than we need,” to the government, “But *we* need to make it easier.” Likewise, in Example (15), three first-person forms appear to refer to three separate referents: “we” seems to refer to the government, yet “our” (in “*our* labeling”) seems to refer to food marketers/manufacturers, whereas “us” refers to the consumer. In this sense, the policy paper is heteroglossic, as the referent of the first-person plural forms “we” and “our” switches seamlessly between the government and the nation, where the latter represents a collective identity incorporating everyone living in the country. Pronouns are, as van Dijk (1998) argues, one of the most effective grammatical categories for expressing and manipulating social relations, status, and power. A similar phenomenon is observed by Mulderrig (2017) in her analysis of childhood obesity health promotional material, in which uses of “we” could be characterized by referential slippage, whereby the perspectives of the government and the texts’ audiences could be conflated (p. 471). A consequence of the semantic vagueness of “we,” Mulderrig (2017) argues, is that “it can construct social groupings, project shared values, assume common goals, and obfuscate responsibility for actions and claims” (p. 471). In the *Tackling Obesity* policy paper, the semantic slippage in uses of “we” to refer to the population and the government allows the latter to “claim a shared voice and perspective with the public,” simultaneously appearing to assume responsibility for the population’s health while imparting that responsibility onto the population at the same time.

In some cases, the social actors and organizations responsible for producing and marketing food and drink products are mentioned explicitly. In such cases, they are consistently presented positively, for example, as being “understanding” of the challenges involved in eradicating obesity and as “leading the way” in measures to this end.

(26) We know many businesses understand this and are leading the way in displaying calorie information, recognizing the demand from their customers.

They are also positively appraised as being adaptable and, as the example below shows, presented as consistently providing (i.e., “*continue* to provide good value”).

(27) We know businesses can adapt and continue to offer customers good value for money but with an emphasis on healthier foods.

Making “healthy” choices in the face of food and drink advertising is not the only thing for which citizen-consumers are responsibilized, as the text also problematizes the “expos[ure]” of children to these advertisements, rather than the advertisements themselves. From this view, it could be argued that it is the role of parents to limit or somehow moderate their children’s engagement with such content, as exemplified below.

(28) Research shows that exposing children to these adverts can increase the amount of food children eat and shape their preferences from a young age.

We also note the use of *preferences* as a synonym of choice, which again maintains focus on the citizen-consumer (here imagined as future food consumers). And this is the discourse that underpins all representations of food and drink marketers—that it is the responsibility of individuals to make good choices in the face of factors, including advertising, which contribute to obesogenic environments. This position is best summarized in the following passage which, while beginning by appearing to acknowledge that obesity is not driven by individual factors alone, then nevertheless proceeds to underscore the role of “choice” by mentioning it 3 times in a single sentence.

(29) Tackling obesity is not just about an individual’s effort, it is also about the environment we live in, the information we are given to make choices, the choices that we are offered, and the influences that shape those choices.

This section has described how individuals are construed as citizen-consumers who are responsible for both developing and eradicating obesity through the *choices* that they make with respect to the food and drink that they purchase and consume. From this position, the role of the government in addressing what it perceives to be problematic rates of obesity in the country is to provide its citizen-consumers with the necessary “knowledge” and “information” with which to make “healthier” choices. In the next section, I examine in more detail how this text presents the role of the government in this process.

### Constructing Government: Helping You to Help Yourself

Now I want to focus on the actions that the government is presented as carrying out, namely, as part of the policies proposed by the text. For this part of the analysis, it is useful to pay particularly close attention the actions and processes, denoted mostly through verbs, which accompany the first-person plural pronoun, *we*, which is the most frequent government-referring word in the text. Doing so brings to the fore evidence of processes that are vague and future actions or so-called “managed actions” (Mulderrig, 2011) but which in each case are positively evaluated.

Examples (30) to (33) exemplify the government’s use of vague language when representing its actions with respect to the obesity policies being introduced.

(30) We need to increase the frequency of these types of interventions for obesity in primary care, and we will be bringing forward a program with incentives for GPs and referral pathways into weight management services in every local health care system.
(31) This effort is an important part of the ambitious action we all need to take to turn the tide on obesity.
(32) Today we are announcing a new set of policies that starts to change this environment.
(33) We are trying to help make a difference. We will continue to follow the evidence and consider what more we can do.

In these examples, the government is presented as carrying out vague processes which have unspecified outcomes, such as “trying to help make a difference” and “consider[ing] what more we can do” (Example (33)), as well as being nominalized as “effort” and “ambitious action” in which agency shifts from the government to all of society (Example (31)). The targets and outcomes of these actions can also be vague, such as “start[ing] to change this environment” in Example (32), which captures a broad range of social and economic factors, and the metaphorically framed but underspecified outcome of “turn[ing] the tide on obesity” (Example (31)).

Another feature of many of the actions (self-)attributed to the government is that they are situated in the future. For example, above the set of policies “*starts to change* this environment” (Example (32)). This is also achieved through certain grammatical choices, particularly the recurring use of verb phrases in the future continuous tense. For example, it was described above how the government “*will be* bringing forward a programme” (Example (30)). The “Obesity Policy in the United Kingdom (in a COVID-19 Context)” section of the text presents a bullet point list of actions prefaced by the phrase “we will be,” where the future actions represent more processes which are framed in the future continuous, including “*introducing* a new campaign,” “*working to* expand weight management services,” “*publishing* a 4-nation public consultation,” “*introducing* legislation,” “*consulting on* our intention,” “*legislating to* end the promotion of foods high in fat,” “*banning* the advertising of HFSS products,” and “*holding* a short consultation.” I would argue that the use of the future continuous tense here expresses a reduced or “hedged” level of commitment to the processes being described. Specifically, the actions that the government pledges are not only situated in the future (i.e., “we *will be* . . .”) but, by being presented as continuous or ongoing (i.e., “we will be *introducing*”), place the focus of the commitment on the action itself as opposed to the result of it. In other words, I would argue that a more committed way to express these pledges would be to either situate the actions in the present (e.g., “we *are* introducing . . .”) or to foreground the completed actions or the outcomes (e.g., “we will *introduce* . . .”). Accordingly, the use of future continuous tense enables the voice of the policy paper to express a relatively restricted level of commitment to the—as already seen, minimal—actions that the government has itself committed to undertake to reduce the country’s rates of obesity, where most of these actions are, instead, to be undertaken by citizen-consumers themselves.

It is also worth commenting briefly upon the use of the list format for presenting the processes that the government is committing to undertake. Ledin and Machin (2015) argue that elements presented in a list are represented as being of the same order, as equal, and as belonging to a common paradigm (p. 7). They argue that

The items in the list are part of the same paradigm but are not connected causally. Simply the list has developed historically for this very purpose, to have these affordances [. . .]; they separate, abstract and reify the items in a paradigm. (Ledin & Machin, 2015, p. 469)

By presenting the government’s actions as a bulleted list, then, a sense of equality and mutual exclusivity is created between its elements. They are presented as separate actions which share a logical consistency in that they all contribute to the same aim or goal—that is, reducing rates of obesity in the country. However, the precise relationships between these elements, and exactly how each will contribute to this shared overarching goal, are matters that are not explained in the article.

Another characteristic of the processes (self-)attributed to the government is, as noted, the construction of “managed actions.” *Managing actions* is a type of verbal process, described by Mulderrig (2011), which

discursively enact[s] a more subtle or “soft” coercive force in contemporary governance [by] (i.) constructing a more indirect form of agency, recasting the government as an “enabling” force, which (ii.) assigns greater autonomy and direct agency to a diverse range of actors, while at the same time, (iii.) specifying desired outcomes and in some cases, (iv.) securing compliance by *assuming volition* on the part of managed actors. (p. 63)

The most notable example of a managed action in this policy paper is arguably in its title, which contains the clause, “Empowering adults and children to live healthier lives,” while Section 4 of the report is titled, “Empowering everyone with the right information to make healthier choices.” More examples of managed actions in the data are given below.

(34) We will also look at ways to make it easier for those struggling with their weight to be referred to specialist support that can help people lose weight and keep it off.
(35) Supporting people to achieve a healthier weight will be crucial to keeping people fit and well as we move forward.
(36) The campaign aims to reach millions of people who need to lose weight, encouraging them to make behavior changes to eat better and move more to prevent or delay the onset of serious diseases.
(37) We must take action to help everyone—adults and children alike to prevent obesity developing. But for adults who are already overweight or living with obesity, we need to do more to support them to reduce their weight and to improve their health.
(38) Helping people to achieve and maintain a healthy weight is one of the most important things we can do to improve our nation’s health.

According to Mulderrig (2011), managing actions “help construct particular relations of power between the government and other social actors. Compared with simple imperatives, Managing Actions construe a reduced or ‘softened’ agency for the government and a corresponding increase in agency (and autonomy) for others” (p. 51). Mulderrig (2011) also describes managed actions as a “key discursive resource in contemporary governance”; she expands that “[f]ar from being merely ‘in vogue’ rhetoric, these forms help organize lines of obligation and responsibility in quite systematic ways” (p. 52).

Examples (34) to (38) also demonstrate some of the ways in which the (managed) actions of the government are legitimated in the policy paper. This includes presenting the actions (and, accordingly, individuals’ weight loss) as necessary by evaluating these as *crucial* (Example (35)) and *important* (Example (38)) and by presenting such actions through use of the modal verbs *need* (Examples (36) and (37)) and *must* (Example (37)), which foreground their necessity.

Another way in which the policies presented are legitimized is through the government’s self-attribution of a range of mental and perceptive verbs to convey the sense in which they, for example, understand, know, and hear of the public’s supposed demand for the policies they are proposing. An example is this statement which responds to an acknowledgment that making “healthy” choices is difficult because of the persuasive power of advertising.

(39) We understand this. We have heard from people up and down the country who want to help themselves. But we have also heard that there are some things where they need our help.
(40) We know that people would welcome more support to make healthier choices for themselves and their families when eating out.

In this example, and there are others in the text, the government is presented as a sympathetic and perceptive social actor who “understand[s]” and “hear[s]” the public whose desires conveniently align with the objectives of their managed actions—that is, they want the government to help them help themselves. Such statements are characteristically vague, like this one, and lack the precise kinds of reference and citations to research that can be found at other points in the policy paper. For example, in Example (21), it was stated that “*Many people* have tried to lose weight but struggle in the face of endless prompts to eat.”

Another legitimation strategy evidenced in these examples is the equivalence that is constructed, both implicitly and explicitly, between, on one hand, obesity and serious illness (Example (36)) and, on the other hand, thinness with fitness, wellness, and health (Examples (35), (37), and (38)). Such cases betray a conflation of thinness with health ideology that implicitly rejects the notion that one can be “fat but fit”—again a contested knowledge terrain in debates about obesity (Ortega et al., 2018).

A subtler legitimation strategy demonstrated in these and other examples presented here is the use of a spatial metaphor which frames the policies (and individuals’ weight loss actions that follow from these) in terms of forward movement. For example, in Example (30), the government represents itself as “*bringing forward* a program,” whereas in Example (35), its managed actions are presented as “crucial to keeping people fit and well *as we move forward*.” This metaphor is vague, in the sense that the (metaphorical) destination into which *we* are moving is unspecified, as is the time frame within which this movement takes place. However, it is a metaphor that helps to positively evaluate and thus legitimize the policies being presented, as the notion of forward movement connotes progress and a sense of advancement or betterment, as has been observed in previous studies of metaphorical language in political genres (e.g., Partington, 2007).

The final set of representations I want to consider also arguably contributes to the legitimation of the policies presented in the text. In particular, I found evidence of the kind of “corporate boast” rhetoric described by Sauntson and Morrish (2011). For instance, in Example (40), the government describes itself as being “really proud” of its “traffic light” food labeling scheme which evaluated as “popular” and described as having been adopted in other countries.

(41) We are really proud of the success of the United Kingdom’s voluntary “traffic light” scheme, which was introduced jointly by the U.K. government and devolved administrations in 2013. The scheme has proved popular with consumers and has been adopted by a majority of manufacturers and retailers. Other countries round the world have followed suit so we want to make sure that our scheme continues to meet the needs of U.K. shoppers and reflects the latest evidence on what works best to help people make healthier choices.

Such self-congratulatory rhetoric may feel out of place in a policy text. However, it performs an important legitimatory function, as it helps to establish the government’s credibility on this issue and demonstrable popularity and success of a scheme that it seeks to expand in this latest package of policies. The notion of the United Kingdom being world-leading in food labeling practices invokes a nationalistic kind of discourse that is consistent with the representations, seen earlier, of the government responding to obesity rates in the interests of the health of the “nation”/”country.” This construction of the United Kingdom as world-leading on this issue is even more explicit in the following passage, in which the country is framed as “help[ing] other countries” to achieve the levels of success that the United Kingdom has purportedly had.

(42) Domestically we have had great success through the Soft Drinks Industry Levy in reducing sugar consumed from soft drinks, and we should help other countries achieve similar gains.

As well as functioning as a legitimation strategy, such passages also evidence the propensity for obesity to be used as a political football—with the reported success of past policies attributed to the government and measured in terms of their “popular[ity]” and other countries adopting similar policies. This political perspective is also evident in the following example, in which the proposed changes to labeling practices—again formulated in nationalistic terms as being “best for Britain”—are framed as having been enabled by the country’s departure from the European Union (EU).

(43) We have an opportunity now we have left the EU to make decisions on labeling which are best for Britain.

We might note that this assertion is inconsistent with the previous passages, in which the policy paper claims that measures that the British government introduced, while inside the EU, have been adopted by other countries. However, this proposition is consistent with the government’s wider pro-Brexit stance.

## Discussion

The analysis presented in the previous section identified a set of textual representations surrounding the things, people, and processes involved in the government’s latest anti-obesity strategy and linked these representations to three overarching discourses: (a) the conceptualization of obesity as a threat that needs to be countered, (b) the construction of citizen-consumers as responsible “choice-makers,” and (c) the construction of the government as a benevolent social actor that helps citizen-consumers to help themselves. These discourses are, of course, related. Beginning with the first discourse, it is through (a) the text establishes obesity as a threat to the health of the country and its healthy system. This threat is also represented as having been intensified by COVID-19, with actions directed at addressing obesity accordingly rendered more urgent. These actions are the subject of discourse (b), which comprises a set of representations which position the public (including imagined readers of the text) as citizen-consumers who are responsible for preventing and eradicating obesity through their lifestyle choices, particularly with regard to the food and drink that they buy and consume. According to this discourse, rates of obesity can be reduced (and the health of the nation and its health service preserved) if citizen-consumers can be educated with the right information to support them to “resist temptation” and make “healthy” food choices in a context characterized by persistent advertising and promotion of nutritionally poor foodstuffs. This is where the government comes in, as discourse (c) comprised a series of representations of the government as a benevolent entity which managed the actions of citizen-consumers by providing them with more information to theoretically enable them to make “healthier” choices in the future—essentially, helping citizen-consumers to help themselves. In this context, the government’s own characterization of the policy text as a “call to action” can be viewed as an accurate one, because the measures described essentially involve the government (as represented by the voice of the policy paper) imploring its citizen-consumers to assume responsibility for their health and the health care system by eradicating their risk of obesity by making “healthier” choices.

Taken together, the discourses identified in the *Tackling Obesity* policy can be viewed as being underpinned by and as espousing a neoliberal framework of governance and public health management. Indeed, the discourses identified, and many of their underlying representations, echo the types of discourses that have been reported in previous studies highlighting the neoliberal framing of obesity, for example, in public health and media texts that were described in the “Obesity Policy in the United Kingdom (in a COVID-19 Context)” section of this article. Yet, in the context of the *Tackling Obesity* policy paper, this discourse feels somewhat insidious. The text acknowledges that “[t]ackling obesity is not just about an individual’s effort.” However, rather than do this in any explicit way, the policy paper instead slips back into employing the more subtle, though increasingly familiar, neoliberal, responsibilizing discourses of self-governance. In this way, I would argue that this policy paper constitutes an example of “lifestyle drift,” which in the context of health promotion can be defined as “the tendency for policy to start off recognizing the need for action on upstream social determinants of health inequalities only to drift downstream to focus largely on individual lifestyle factors” (Popay et al., 2010, p. 148).

Neoliberal modes of governance and the discourses through which they are enacted are, as noted, by now familiar, particularly to critical scholars interested in the discursive dynamics of public health. As discussed at the beginning of this article, neoliberalism has been the guiding principle of government in the United Kingdom for around four decades (at the time of writing). As such, the neoliberal discourses that pervade the *Tackling Obesity* policy paper are not new but rather represent the latest step in what Hall (2011) describes as “the long march of the neo-liberal revolution” (p. 705). The neoliberal principles underpinning this recent policy paper therefore do not come as much of a surprise, though they may give us cause to be skeptical of the likely success of this policy initiative. This is because if one accepts the premise of the policy paper, that obesity constitutes one of the “greatest challenges” faced by this generation (as the policy paper itself claims), then one must also accept the possibility (likelihood) that it is the neoliberal policies that have predominated over the last 40 or so years that have brought us to this point of purported “crisis.”

The limitations of neoliberalism have been widely theorized (Harvey, 2005), including in relation to obesity (Brookes & Baker, 2021) and public health more broadly (Brookes & Harvey, 2015). One of the most cited limitations of neoliberal policies is that they overlook or ignore the fact that, when people engage in behaviors that are damaging to their health, this may be due to the constraints of their life circumstances rather than lack of awareness. In such cases, simply raising awareness of the riskiness of certain behaviors is not sufficient to instigate behavior, as it does not reduce or remove those constraints that give rise to such “unhealthy” behaviors in the first place. Indeed, a now substantial body of evidence exists that obesity is a multifaceted and complex health issue in which individuals’ lifestyles are just one of many factors (Butland et al., 2007). Yet, because lifestyle drift leads back to a focus on individuals, it often precludes the possibility that these many social, political, and economic factors are acknowledged or addressed (Godziewski, 2021). Indeed, the foregoing analysis has demonstrated that factors outside of individual choice which have been found to contribute to the development of obesity—such as the high cost of fresh and nutritious foodstuffs, the lower cost and aggressive promotion of nutritionally poorer alternatives, and the design of urban spaces which makes them more conducive to driving than walking or cycling—are largely elided from the policy paper. When such factors are hinted at, such as the role of food and drink manufacturers and marketers, the precise roles of these organizations in contributing to obesity are backgrounded or mitigated, being more likely to be presented positively on the rare occasions that they mentioned explicitly. This approach is consistent with the pro-business and economically liberal ethos of the governing Conservative party. However, it is worth bearing in mind that public health experts have expressed the view that public health policies around obesity should be engaging with these stakeholders directly and explicitly (Boswell, 2020).

Another aspect of the neoliberal discourses espoused by the *Tackling Obesity* policy paper is that it positions the public as informed and “rational” citizen-consumers who, if they can only harness more information and knowledge with respect to obesity and risk, will then act in the interests of their health and for the good of the state and its health care system. Indeed, Brown and Baker (2012) argue that a “theme” in the policy of responsibilization is

for the state to propagate risk knowledge with the aim of increasing the individual capacity for what the state deems as responsible free choice. This knowledge in turn should be employed by the citizenry to guide their individual conduct. Thus, campaigns to introduce more legible food labelling schemes so that shoppers can see at a glance whether the product is low, medium or high in fat, sugar and salt [. . .] are intended to place more risk knowledge at the disposal of the consumer. Governmental campaigns and advice on how to maintain good health can be seen as this kind of responsibilization, the propagation of this sort of knowledge emphasizing the individual’s “duty to be well” . . . (p. 20)

We can clearly see such an approach at work in the *Tackling Obesity* policy paper. Yet, this approach could prove problematic, as it neglects to consider the plethora of factors *other than* health and nutrition which have been found to influence individuals’ food choices (Ajzen, 1991), as well as a presupposing levels of literacy and numeracy skills which, where lacking, could result in a widening of health inequalities (Gilbert et al., 2018). There are other gaps in the policy paper which have, as noted, been pointed out by various political commentators, such as efforts to make the “healthier choices” that the paper urges citizen-consumers to make more affordable and accessible. It is beyond the objective of this article to explore the substance of the policy in much depth, though the backgrounding of these and other factors is clearly important to the foregrounding of individuals’ lifestyle choices in the text.

It is also important to consider the implications that the “lifestyle drift” toward a neoliberal set of obesity policies may have for not only the physical but also emotional well-being of people with obesity (and the public generally, for that matter). A widely acknowledged corollary of neoliberal models of public health is that the foregrounding of individual choices means that individuals are presupposed to be responsible both for developing and eradicating obesity. This means that if they are unsuccessful in either of these, they are liable to be blamed and, accordingly, stigmatized. Indeed, the notion that calorie labeling is the “key” to making “healthy” choices, as the policy paper asserts, presupposes that the provision of such information (as the policy package promises) will remove the credible reasons why individuals may not make the right choices and so develop obesity. In other words, if food labeling is presented as the “key” to weight loss, then the provision of better food labeling means that failure to lose weight will be the fault of the individual. More specific representations contributing to the discourses of this policy paper may also intensify weight stigma. For example, militaristic metaphors, which frame obesity as a “war” or weight loss as a “struggle”, have the potential to morph into wars on people with obesity and their lifestyles (Herndon, 2005).

As part of a broader discourse which positions obesity as a threat to society, this analysis has also demonstrated how obesity and people with it were represented in the policy paper as being a burden on the NHS. As with the other responsibilizing discourses observed in this text, this involved a balance between, on one hand, presenting people with obesity as relying heavily on health care services and, on the other hand, obscuring other factors which are likely to have contributed to the problems faced by the NHS, such as ineffective health care policy and chronic underfunding of it by successive governments (see Baker et al., 2019; Brookes & Harvey, 2016b). Raising the stakes for people with obesity, by adding to them the status of the NHS (e.g., in statements such as “we owe it to the NHS to move toward a healthier weight”), is thus likely add more unhelpful pressure to people who, as the policy paper itself puts it, already “struggle to lose weight.” Yet, the stakes are raised even further when the policy paper tells us that “Tackling obesity would reduce pressure on doctors and nurses in the NHS, and free up their time to treat other sick and vulnerable patients” and that “you can play your part to protect the NHS and save lives.” Thus, citizen-consumers with obesity are told to lose weight not only for their own health but also to “protect” the NHS and save the lives of others. This could contribute to public discourses which blame and shame people with obesity for the problems faced by the NHS, including waiting times and lack of treatment access, as well as for cases where these problems result in people suffering more severe health complications.

Weight stigma can have adverse consequences for individuals’ physical and emotional well-being. It can lead to unhealthy bodily practices, including crash dieting and the development of eating disorders (Puhl & Suh, 2015), and contribute to internalized shame and forms of mental distress, including mental illnesses (see Tomiyama et al., 2018, for a review of literature on this topic). There is evidence that the contexts surrounding the COVID-19 pandemic may compound these impacts, as rates of mental illnesses like anxiety and depression have increased markedly (Salari et al., 2020), while early studies of media coverage of obesity in the COVID-19 context point to an intensification of stigmatizing representations (Brookes, 2021). There is a worrying irony that the pandemic, which is the motivation for this latest policy initiative, may compound any ill-effects that arise from its presentation.

## Conclusion

In this article, I have critically examined the discourses that constitute the *Tackling Obesity* policy paper, linking these to underlying ideologies around obesity, citizenship, government, and public health. I have argued that the representations of the things, people, and processes linked to this policy issue contribute to three broad discourses which operate in tandem to problematize obesity in the United Kingdom, position the public as responsibilized citizen-consumers, and then present the government, alongside food and drink industry stakeholders, as a benevolent social actor whose actions are intended to “help” individuals to make “healthier choices.” I have argued that these discourses are underpinned by and propagate a neoliberal framework of public health governance, but that the ways in which these policies are presented resembles the process of “lifestyle drift,” whereby the policy is presented in a way which gives the initial impression that it will address social and economic determinants of ill-health before ultimately neglecting such aspects in favor of focusing on individual lifestyle factors. Although it is beyond the scope of this article to comment in depth on the substance of the policies themselves, I have suggested that we may have cause to be skeptical about their likely success, given that the neoliberal modes of governance that have predominated in the United Kingdom over the last 40 or so years have brought us to this point of purported “obesity crisis.” The effectiveness (or otherwise) of these policies will become clearer once they have been fully implemented. However, the discourses that characterize their introduction in the *Tackling Obesity* policy paper—which responsibilize people with obesity for their health, the health of the NHS, and the health of other people in the country—may lead to further stigmatizing of people with obesity. Moreover, I have argued that this stigmatization may be intensified by the very pandemic context that has motivated this new set of policies, which may only exacerbate the physical and emotional turmoil caused by weight stigma and shaming.

A rebuttal to the analysis presented in this article could be that the presentation of any policy surrounding a complex health issue like obesity will inevitably involving collapsing a number of elements, with some elements being foregrounded while others are backgrounded. This is, to an extent, a valid defense. Yet, it also brings into sharp focus the fact that the creation of this text has involved the selection of certain factors to be foregrounded and other backgrounded for the presentation of a coherent and digestible policy package. The foregrounding of individual lifestyle factors, at the expense of addressing most (all?) other factors that contribute to the complexity of obesity, is, I would argue, a motivated discursive choice, and one which serves the neoliberal doxa according to which this and other U.K. governments have ruled. The public may be better served by a more balanced set of public health discourses, which seek to balance the importance of lifestyle factors with the influence of social and economic health determinants. Such discourses may at least better reflect the true complexity of this social justice issue. Whatever the case, it is crucial to engage closely and critically with public health policy presentation, in texts such as the *Tackling Obesity* policy, as the representations, discourses, and underlying ideologies that characterize such documents can help to uncover the assumptions that have informed and driven such policies, as well as, crucially, the ways in which the public will interpret them. I add my voice to those such as Fairclough (2013) and Mulderrig (2019a, 2019b) to argue that CDA offers a rigorous set of methods and concepts for critically examining such texts, being based on scrutinizing text producers’ linguistic and grammatical choices and considering the effects of these. In this sense, CDA provides a useful methodological toolkit for developing and enacting critical language awareness in the domain of public health rhetoric.
